# Supplementary material for: Molecular Analysis of Biliary Tract Cancers with the Custom 3′ RACE-Based NGS Panel
Source: Diagnostics (Basel). 2023 Oct 10;13(20):3168. doi: 10.3390/diagnostics13203168 (PMC10605186; doi:10.3390/diagnostics13203168)
Supplement: Supplementary file 1 [file diagnostics-13-03168-s001.zip › diagnostics-2617903-supplementary.pdf]

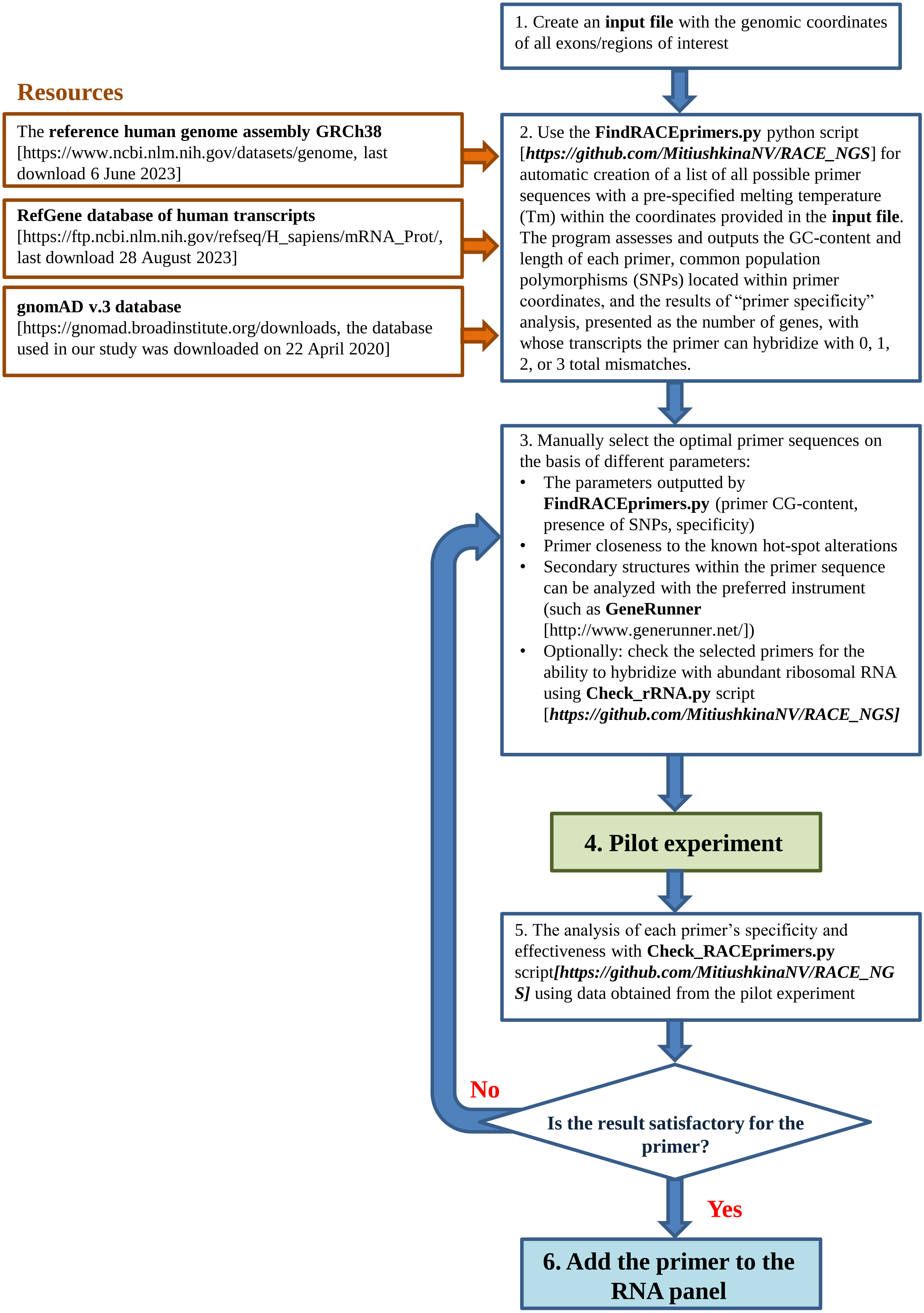

Figure S1. The scheme shows the process used to design the targeted RNA panel on the basis of the 3’ RACE approach

R1 and R2 .fastq files generated by NGS sequencing of libraries prepared with 3’ RACE-based approach

Analysis of translocations, involving the FGFR1-4 genes was performed with the **STAR-Fusion v1.10.1** package [https://github.com/STAR-Fusion/STAR-Fusion] [33].

The manual filtering of the outputted variants was performed as follows:

- Only rearrangements supported with at least 10 different reads were kept;
- The kinase domain of the respective FGFR-gene should not have been affected by rearrangement.

Preprocessing of files, required for the analysis of mutations and gene expression:

1. Extracting the pseudo-UMI sequences from the distal part of random primer used in the reverse transcription reaction with **UMI-tools v. 1.1.4** (*--bc-pattern NNNNNNNN*) [https://github.com/CGATOxford/UMI-tools] [35].
2. Alignment of reads using the **STAR program v2.7.8a** [https://github.com/alexdobin/STAR] [36]. The two random nucleotides located between the adapter and primer sequences, as well as the 8 nucleotides remained after extraction of “UMIs” were clipped by using the following parameters: *--clip5pNbases 2,2 --clip3pNbases 8,0*.
3. Indexing the **.bam** files with samtools v1.17 [https://github.com/samtools/samtools].
4. Removing PCR and optical duplicates using the **UMI-tools dedup** command.
5. Sorting the **.bam** files by *queryname* with the **Picard toolkit v3.0.0** [https://broadinstitute.github.io/picard/].
6. Applying the custom python script **Filter_RACEbam.py** [https://github.com/MitiushkinaNV/RACE_NGS] to filter out improperly aligned/unspecific reads and create the catalogue of reads, generated from each primer used at the PCR-enrichment step during the library preparation (**_filter.txt** file).
7. Sorting the filtered .bam file from the previous step by *coordinate* and create index using the **Picard toolkit.**

Analysis of gene expression was done using custom python script **Expression_count.py** [https://github.com/MitiushkinaNV/RACE_NGS]. This script counts the number of reads within regions, provided in a separate input file. For the analysis we selected the 15bp-long regions, located at the 90bp distance from the 5’ end of the primers, used for enrichment, inside the next exon (illustrated below). The single **.tsv** file was created for all analyzed samples.

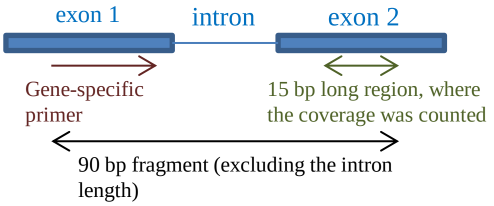

The table with expression counts, generated in the previous step, was analyzed with the **R software v4.1.1** [https://www.R-project.org/] [41]. The expressions of all genes of interest were normalized relative to the expression of the three referee genes (DDX23, GOLGA5 and SEL1L), included in the targeted panel. Then, the bioclinical associations were studied as described in the manuscript.

Calling and annotation of mutations:

1. The calling of mutations in all samples was done using the custom python script **RACE_caller.py** [https://github.com/MitiushkinaNV/RACE_NGS]. The 5% alternate allele fraction (*--min_alt*) was used as a threshold.
2. The **ANNOVAR** software [https://annovar.openbioinformatics.org] [39] was used to annotate the called genetic variants using the data from the Ensembl genome database [https://www.ensembl.org], the gnomAD database [https://gnomad.broadinstitute.org], the COSMIC [https://cancer.sanger.ac.uk/cosmic] and the ClinVar databases [https://www.ncbi.nlm.nih.gov/clinvar].

The manual filtering of the called genomic variants was performed as follows:

- All synonymous nucleotide changes and variants with the population allele frequency of 1% or higher (according to the gnomAD database) were filtered out;
- The variants with population allele frequency $\geq$ 0.01% were also considered likely to be rare germline SNPs and filtered, if the alternate allele fraction was close to 0.5 and total occurrences in the COSMIC database was less than or equal to 30;
- The mutations with total occurrences in COSMIC less than or equal to 30 (meaning, they are not hot-spot variants), which were present 3 or more times in our collection of BTCs, where considered to be recurrent artifacts and filtered;
- The mutations with total occurrences in COSMIC less than or equal to 30, and the mutant allele fraction less than 10% or the total number of reads with alternate allele less when 10 were considered to be random artifacts and filtered;
- In samples with high number of called variants, even hot-spot mutations with low allele fraction could be filtered out (the exact threshold depended on the allelic fractions characteristic for the other variants called in this sample).

Figure S2. The scheme of bioinformatics analysis of data obtained using 3’ RACE-based targeted RNA sequencing

Table S1. Primers for PCR-enrichment included in the 3' RACE-based NGS panel for BTCs

#	Gene	Exon	Transcript	Primer name	Primer sequence
1	FGFR1	12	ENST00000447712.7	FGFR_P20	CCCTACACGACGCTCTTCCGATCTNNCAGACCTGATCTCAGAAATGGAG
2	FGFR1	14	ENST00000447712.7	FGFR_P21	CCCTACACGACGCTCTTCCGATCTNNTGATGAAGATAGCAGACTTTGGC
3	FGFR1	17	ENST00000447712.7	FGFR_P84	CCCTACACGACGCTCTTCCGATCTNNTCACAGAGACCCACCTTCAAG
4	FGFR1	18	ENST00000447712.7	FGFR_P24	CCCTACACGACGCTCTTCCGATCTNNATTCCGTCTTCTCTCATGAGCC
5	FGFR1	18	ENST00000447712.7	FGFR_P25	CCCTACACGACGCTCTTCCGATCTNNGACCAGTACTCCCCCAGCTTT
6	FGFR2	5	ENST00000358487.10	FGFR_P96	CCCTACACGACGCTCTTCCGATCTNNCATACTGGACCAACACAGAAAAGA
7	FGFR2	6	ENST00000358487.10	FGFR_P3	CCCTACACGACGCTCTTCCGATCTNNTCACACGTACCACCTGGATGTT
8	FGFR2	7	ENST00000358487.10	FGFR_P4	CCCTACACGACGCTCTTCCGATCTNNTGTCTGCAAGGTTTACAGTGATG
9	FGFR2	7	ENST00000358487.10	FGFR_P5	CCCTACACGACGCTCTTCCGATCTNNCAAATGCCTCCACAGTGGTCG
10	FGFR2	9	ENST00000358487.10	FGFR_P7	CCCTACACGACGCTCTTCCGATCTNNCGCCTGGAAGAGAAAAGGAGATT
11	FGFR2	12	ENST00000358487.10	FGFR_P26	CCCTACACGACGCTCTTCCGATCTNNAAAGACCTTTCTGATCTGGTGTC
12	FGFR2	14	ENST00000358487.10	FGFR_P27	CCCTACACGACGCTCTTCCGATCTNNGACTCGCCAGAGATATCAACAATA
13	FGFR2	16	ENST00000358487.10	FGFR_P28	CCCTACACGACGCTCTTCCGATCTNNGACACAGAATGGATAAGCCAGC
14	FGFR2	17	ENST00000358487.10	FGFR_P29	CCCTACACGACGCTCTTCCGATCTNNGCAGTTGGTAGAAGACTTGGATC
15	FGFR2	18	ENST00000358487.10	FGFR_P8	CCCTACACGACGCTCTTCCGATCTNNCCAGACCCCATGCCTTACGAA
16	FGFR2	18	ENST00000358487.10	FGFR_P9	CCCTACACGACGCTCTTCCGATCTNNGAAGTTCTTGTTCTTCAGGAGATG
17	FGFR3	6	ENST00000440486.8	FGFR_P97	CCCTACACGACGCTCTTCCGATCTNNGGAGAACAAGTTTGGCAGCAT
18	FGFR3	8	ENST00000440486.8	FGFR_P11	CCCTACACGACGCTCTTCCGATCTNNTTTCTCATCACTCTGCGTGGCT
19	FGFR3	12	ENST00000440486.8	FGFR_P12	CCCTACACGACGCTCTTCCGATCTNNGTGTCTGAGATGGAGATGATGAA
20	FGFR3	14	ENST00000440486.8	FGFR_P13	CCCTACACGACGCTCTTCCGATCTNNGTGCACAACCTCGACTACTACA
21	FGFR3	16	ENST00000440486.8	FGFR_P85	CCCTACACGACGCTCTTCCGATCTNNCCAACTGCACACACGACCT
22	FGFR3	17	ENST00000440486.8	FGFR_P18	CCCTACACGACGCTCTTCCGATCTNNGACCTGGACCGTGTCCTTACC
23	FGFR3	18	ENST00000440486.8	FGFR_P14	CCCTACACGACGCTCTTCCGATCTNNCAGGGGACGACTCCGTGTTTG
24	FGFR4	9	ENST00000292408.9	FGFR_P31	CCCTACACGACGCTCTTCCGATCTNNATACGGACATCATCCTGTACGC
25	FGFR4	12	ENST00000292408.9	FGFR_P32	CCCTACACGACGCTCTTCCGATCTNNTCGGCCGACACAAGAACATCAT
26	FGFR4	14	ENST00000292408.9	FGFR_P33	CCCTACACGACGCTCTTCCGATCTNNCGGCGTCCACCACATTGACTA
27	FGFR4	17	ENST00000292408.9	FGFR_P86	CCCTACACGACGCTCTTCCGATCTNNTCCCAGAGGCCTACCTTCAA
28	FGFR4	18	ENST00000292408.9	FGFR_P35	CCCTACACGACGCTCTTCCGATCTNNGCTCCTCCAGCGATTCTGTCTT
29	FGFR4	18	ENST00000292408.9	FGFR_P36	CCCTACACGACGCTCTTCCGATCTNNTACCTCGACCTCCGCCTGAC
30	IDH1	4	ENST00000345146.7	FGFR_P52	CCCTACACGACGCTCTTCCGATCTNNCTTGTGAGTGGATGGGTAAAACC
31	IDH2	4	ENST00000330062.8	FGFR_P88	CCCTACACGACGCTCTTCCGATCTNNCAAAAACATCCCACGCCTAGTC
32	IDH2	4	ENST00000330062.8	FGFR_P54	CCCTACACGACGCTCTTCCGATCTNNGTGGAAAAGTCCCAATGGAACTAT
33	ERBB2	7	ENST00000269571.10	FGFR_P45	CCCTACACGACGCTCTTCCGATCTNNGCCCTGGTCACCTACAACACA
34	ERBB2	8	ENST00000269571.10	FGFR_P46	CCCTACACGACGCTCTTCCGATCTNNAACTACCTTTCTACGGACGTGG
35	ERBB2	16	ENST00000269571.10	FGFR_P47	CCCTACACGACGCTCTTCCGATCTNNCTGTGTGGACCTGGATGACAAG
36	ERBB2	17	ENST00000269571.10	FGFR_P48	CCCTACACGACGCTCTTCCGATCTNNGTGGTCTTTGGGATCCTCATCA
37	ERBB2	18	ENST00000269571.10	FGFR_P49	CCCTACACGACGCTCTTCCGATCTNNAGGTGCTTGGATCTGGCGCTT
38	ERBB2	19	ENST00000269571.10	FGFR_P44	CCCTACACGACGCTCTTCCGATCTNNGAATGTGAAAATTCCAGTGGCCAT
39	ERBB2	19	ENST00000269571.10	FGFR_P87	CCCTACACGACGCTCTTCCGATCTNNCCCCAAAGCCAACAAAGAAATCT
40	ERBB2	21	ENST00000269571.10	FGFR_P51	CCCTACACGACGCTCTTCCGATCTNNGGGATGAGCTACCTGGAGGAT
41	ERBB2	21	ENST00000269571.10	FGFR_P83	CCCTACACGACGCTCTTCCGATCTNNCTGGTCAAGAGTCCCAACCATG
42	PIK3CA	2	ENST00000263967.4	FGFR_P55	CCCTACACGACGCTCTTCCGATCTNNGACCTTCGGCTTTTTCAACCCTT
43	PIK3CA	2	ENST00000263967.4	FGFR_P56	CCCTACACGACGCTCTTCCGATCTNNCGTAAGTGTTACTCAAGAAGCAGA
44	PIK3CA	2	ENST00000263967.4	FGFR_P57	CCCTACACGACGCTCTTCCGATCTNNGAATGATAGTGACTTTAGAATGCCT
45	PIK3CA	5	ENST00000263967.4	FGFR_P89	CCCTACACGACGCTCTTCCGATCTNNGCAACCTACGTGAATGTAAATATTCGA
46	PIK3CA	5	ENST00000263967.4	FGFR_P90	CCCTACACGACGCTCTTCCGATCTNNCCTTTGGGTTATAAATAGTGCACTCAG
47	PIK3CA	6	ENST00000263967.4	FGFR_P91	CCCTACACGACGCTCTTCCGATCTNNTGTGACAATGTGAACACTCAAAGAG

48	PIK3CA	7	ENST00000263967.4	FGFR_P61	CCCTACACGACGCTCTTCCGATCTNNAAGGCCGAAAGGGTGCTAAAGA
49	PIK3CA	8	ENST00000263967.4	FGFR_P92	CCCTACACGACGCTCTTCCGATCTNNAGTATCTGGAAAAATGGCTTTGAATCT
50	PIK3CA	10	ENST00000263967.4	FGFR_P63	CCCTACACGACGCTCTTCCGATCTNNCAGCTCAAAGCAATTTCTACACGA
51	PIK3CA	12	ENST00000263967.4	FGFR_P93	CCCTACACGACGCTCTTCCGATCTNNATTGGCCTCCAATCAAACCTG
52	PIK3CA	14	ENST00000263967.4	FGFR_P65	CCCTACACGACGCTCTTCCGATCTNNGAAGCACCTGAATAGGCAAGTC
53	PIK3CA	19	ENST00000263967.4	FGFR_P66	CCCTACACGACGCTCTTCCGATCTNNGATGCAGCCATTGACCTGTTTAC
54	PIK3CA	20	ENST00000263967.4	FGFR_P67	CCCTACACGACGCTCTTCCGATCTNNGTGCCATTTGTTTTGACACAGGAT
55	PIK3CA	21	ENST00000263967.4	FGFR_P68	CCCTACACGACGCTCTTCCGATCTNNGAATGATGCACATCATGGTGGCT
56	PIK3CA	21	ENST00000263967.4	FGFR_P69	CCCTACACGACGCTCTTCCGATCTNNGCCTTAGATAAAACTGAGCAAGAG
57	PIK3CA	21	ENST00000263967.4	FGFR_P70	CCCTACACGACGCTCTTCCGATCTNNCTGGAATGCCAGAACTACAATCTT
58	PIK3CA	21	ENST00000263967.4	FGFR_P71	CCCTACACGACGCTCTTCCGATCTNNTCGACAGCATGCCAATCTCTTC
59	KRAS	2	ENST00000311936.8	FGFR_P94	CCCTACACGACGCTCTTCCGATCTNNTGAATATAAACTTGTGGTAGTTGGAGC
60	KRAS	3	ENST00000311936.8	FGFR_P76	CCCTACACGACGCTCTTCCGATCTNNCCTGTCTCTTGGATATTCTCGAC
61	KRAS	4	ENST00000311936.8	FGFR_P77	CCCTACACGACGCTCTTCCGATCTNNGGCTCAGGACTTAGCAAGAAGTT
62	KRAS	4	ENST00000311936.8	FGFR_P95	CCCTACACGACGCTCTTCCGATCTNNGGACTCTGAAGATGTACCTATGGTC
63	NRAS	2	ENST00000369535.5	FGFR_P79	CCCTACACGACGCTCTTCCGATCTNNGCTGGTGTGAAATGACTGAGTAC
64	NRAS	3	ENST00000369535.5	FGFR_P80	CCCTACACGACGCTCTTCCGATCTNNGAAACCTGTTTGTTGGACATACTG
65	NRAS	4	ENST00000369535.5	FGFR_P81	CCCTACACGACGCTCTTCCGATCTNNGCCAAGAGTTACGGGATTCCAT
66	BRAF	15	ENST00000646891	FGFR_P82	CCCTACACGACGCTCTTCCGATCTNNTATTTCTTCATGAAGACCTCACAG
67	CD274	5	ENST00000381577.4	FGFR_P43	CCCTACACGACGCTCTTCCGATCTNNCCTTGGTGTAGCACTGACATTC
68	DDX23	9	ENST00000308025.8	FGFR_P37	CCCTACACGACGCTCTTCCGATCTNNCCTAATGGAGAAGAGGCGAACC
69	GOLGA5	6	ENST00000163416.7	FGFR_P41	CCCTACACGACGCTCTTCCGATCTNNCTTGGAGTCCTCTAAGCAGGAAT
70	SEL1L	13	ENST00000336735.9	FGFR_P40	CCCTACACGACGCTCTTCCGATCTNNGAGTAATGAGACAGCTCTCCACT

Table S2. Genomic analysis of 47 iCCA samples by 3' RACE-based targeted RNA sequencing and Illumina TruSight Tumor 170 kit

Patient	Diagnosis	Age	Sex	ERBB2 amplification determined by PCR	MSI (pentaplex panel)	Targeted RNA sequencing results (custom library preparation based on 3'-RACE)	Targeted DNA/RNA sequencing results (Illumina TruSight Oncology 170 panel)	Comment
#1	iCCA	59	f	absent	MSI-	WT	**TP53 R282W (0.557), BRIP1 D149G (0.518), GNAS A210V (0.448),** *GNAQ Y101X (0.077)*	
#2	iCCA	55	m	absent	MSI-	FGFR2-LRRFIP2 (F17;L3) (10575)	FGFR2-LRRFIP2 (1700), **AKT2 R176C (0.514), BAP1 splice site c.376-2A>G (0.479, ClinVar: Likely_pathogenic)**	
#3	iCCA	52	m	absent	MSI-	IDH1 R132L (0.067)	WT	Mutation was missed by targeted DNA sequencing due to its low prevalence (mutant allele fraction < 2%)
#4	iCCA	49	m	absent	MSI-	NRAS Q61R (0.231)	**IDH1 R82K (0.421)**, *NRAS Q61R (0.090), BAP1 splice site c.67+1G>T (0.070), NOTCH3 N1179S (0.069)*	
#5	iCCA	64	f	absent	MSI-	IDH1 R132L (0.468)	**RAD54L A704G (0.694)**, IDH1 R132L (0.247), *CARD11 S622del (0.060), VHL S38C (0.060), FGF19 H33P (0.056), BRAF splice site c.2128-2->T (0.055), TSC2 splice site c.1119+2T>G (0.050)*	
#6	iCCA	61	f	absent	MSI-	IDH1 R132C (0.240)	IDH1 R132C (0.218), PTEN Y174Lfs*6 (0.162) + *I253Tfs*3 (0.073) + K254Q (0.073)*	

#7	iCCA	55	f	absent	MSI-	FGFR2-CFAP57 (F17;C12) (2370), IDH R132C (0.281)	FGFR2-CFAP57 (3711), **NOTCH3 G1462S (0.480)**, BAP1 A321Kfs*13 (0.180), IDH1 R132C (0.144)	
#8	iCCA	42	f	absent	MSI-	FGFR2-AHCYL1 (F17;A2) (5996)	FGFR2-AHCYL1 (59), FLT3 A352T (0.393), *ERBB2 W9G (0.055),* FGFR2 amplification (fold change 3.339), MYC amplification (fold change 2.636), MDM4 amplification (fold change 2.419), NRAS amplification (fold change 2.383)	
#9	iCCA	63	m	absent	MSI-	WT	**BRCA2 H2623R (0.763; ClinVar: Pathogenic/Likely_pathogenic),** SMAD4 splice site c.1447+2T>C (0.357), CDH1 V475M (0.222)	
#10	iCCA	62	m	absent	MSI-	KRAS G12D (0.452)	**TP53 E204X (0.496), KRAS G12D (0.440),** CDKN2A S43Tfs*9 (0.389), APC P1691L (0.214)	
#11	iCCA	69	m	absent	MSI-	BRAF V600E (0.443)	**TP53 V216L (0.764), ATM V27L (0.543)**, **ERBB4 F1102C (0.469), BRAF V600E (0.422),** APC S1223C (0.335), PALB2 L531Cfs*30 (0.328)	
#12	iCCA	63	f	absent	MSI-	NRAS Q61K (0.539), PIK3CA E726K (0.181)	**CSF1R E949A (0.536), CREBBP M866I (0.475), MYCL A87T (0.417),** NRAS Q61K (0.348), PIK3CA E726K (0.141)	
#13	iCCA	67	f	absent	MSI-	WT	**CREBBP A909V (0.403),** MAP2K1 L98_I103del (0.193), HNF1A G288W (0.183), TET2 Q888X (0.174), DNMT3A R882H (0.165), *MYC Q52del (0.051)*	

#14	iCCA	43	f	**present**	MSI-	WT	PIK3R1 E332K (0.241), *FOXL2 A14G (0.086), CREBBP H2384P (0.071), ALK H258D (0.069),* ERBB2 amplification (fold change 6.57), MYC amplification (fold change 2.267)	
#15	iCCA	50	m	absent	MSI-	WT	**KIT V50L (0.473), MUTYH R217H (0.470, ClinVar: Pathogenic/Likely_pathogenic),** NOTCH1 *C48W (0.052)* + C750R (0.324)	
#16	iCCA	61	f	absent	MSI-	IDH1 R132G (0.465)	**BTK E240Nfs*37 (0.742), PMS2 L245V (0.608), AKT2 T431M (0.437), LAMP1 R77H (0.412),** IDH1 R132G (0.292), INPP4B T645R (0.149)	
#17	iCCA	71	m	absent	MSI-	KRAS G12C (0.506)	**HNF1A E48K (0.607),** KRAS G12C (0.271), BAP1 G13V (0.236)	
#18	iCCA	67	m	absent	MSI-	FGFR2-ZMYM4 (F17;Z2) (1711)	FGFR2-ZMYM4 (13023), TSC1 V178I (0.288), FGFR2 amplification (fold change 2.227)	Low coverage of the target exonic regions in DNA sequencing
#19	iCCA	63	f	absent	MSI-	IDH1 R132G (0.304)	**TP53 G105D (0.412)**, TET2 P174H (0.235), IDH1 R132G (0.228), PPP2R2A M358T (0.224)*, CREBBP H2384P (0.078)*	
#20	iCCA	63	m	absent	MSI-	WT	**TP53 E286K (0.630), NF1 R416X (0.436)**, AR Q80_E81insQQQ (0.302), NOTCH1 E694K (0.260), KDR T42K (0.173), FGF4 R134W (0.159), MET A1196E (0.141), *MSH3 P63A (0.08)*	
#21	iCCA	45	f	absent	MSI-	IDH1 R132C (0.378)	IDH1 R132C (0.254), MSH3 splice site c.1568+1G>C (0.124)	

#22	iCCA	64	m	absent	MSI-	IDH1 R132C (0.415)	IDH1 R132C (0.386), *FOXL2 G177S (0.058)*	
#23	iCCA	63	m	absent	MSI-	WT	**ABRAXAS1 A382Sfs*11 (0.583), NBN E585G (0.464), CDK12 R358G (0.412),** NF1 P22S (0.105)	
#24	iCCA	65	f	absent	MSI-	KRAS G12D (0.523), PIK3CA C420R (0.704)	ESR1-CCDC170 (751 reads), **PIK3CA C420R (0.549), NOTCH1 M1580I (0.503),** GNAS ENST00000349036 A436D (0.356), **CTNNB1** S33C (0.181) **+ R225H (0.471)**, KRAS G12D (0.218), ARID1A H203Sfs*197 (0.139), CREBBP Q1765Rfs*6 (0.125)	
#25	iCCA	70	m	absent	MSI-	KRAS G12V (0.446)	**NBN D95N (0.855), NOTCH1 R879Q (0.826), GEN1 T105R (0.596), APC S2365P (0.488),** BAP1 splice site c.123-1G>A (0.338), KRAS G12V (0.313), SMAD4 D2G (0.218)	
#26	iCCA	66	m	absent	MSI-	IDH1 R132G (0.445)	IDH1 R132G (0.165)	

#27	iCCA	61	f	absent	**MSI+**	<u>ERBB2 R678Q (0.427), PIK3CA E542K (0.302)</u>	**ERCC2 K671Qfs*103 (0.501; ClinVar: Pathogenic), ROS1 L2149I (0.471), MLH1 splice site c.589-1G>A (0.439; ClinVar: Pathogenic/Likely_pathogenic) +** L352Yfs*18 (0.203), CDKN2A R87W (0.203) + R107C (0.226), ARID1A Q766Sfs*67 (0.224), GNAS ENST00000349036 P540L (0.203) + R600H (0.124) + R829H (0.180), <u>PIK3CA E542K (0.184),</u> MSH6 F1088Sfs*2 (0.192), KMT2A C2482Y (0.163), TSC1 R420Gfs*20 (0.166), CCND1 R231H (0.148), <u>ERBB2 R678Q (0.145),</u> ERBB3 G284R (0.145), DNMT3A P59Rfs*13 (0.141), ATR A823V (0.131), BARD1 S575N (0.131) + L577Cfs*14 (0.121), FGFR1 R809Q (0.127), PDGFRB F995S (0.111) + *P1070S (0.09),* BRCA2 T3033Nfs*11 (0.108), *APC V1872D (0.099)*	
#28	iCCA	64	f	nd	MSI-	WT	**JAK3 R899Q (0.436),** *MLLT3 S190del (0.06), CREBBP Q2216del (0.058), AR R544S (0.056)*	

#29	iCCA	34	m	absent	MSI-	PIK3CA E545K (0.133)	**MLH1 D41G (0.502; ClinVar: Pathogenic), NOTCH2 S2403I (0.47),** HRAS splice site c.290+2T>C (0.147), *STK11 P281Rfs*6 (0.149*), TP53 R175H (0.129), FGFR2 R251X (0.098) + G484D (0.109), *MUTYH G475W (0.095), PIK3CA R4Q (0.093) + E545K (0.062)*	Mutations in the FGFR2 gene were not identified by targeted RNA sequencing, probably because of low mutant allele expression (these mutations are unlikely to be activating).
#30	iCCA	58	m	absent	MSI	KRAS G12D (0.213)	**FGF5 E235G (0.512), NOTCH2 R2060C (0.507), CHEK2 K312E (0.485),** TP53 P153Afs*28 (0.113), *FLT3 S963L (0.077), NF1 Y628Tfs*3 (0.057), MET Y126C (0.044), KRAS G12D (0.034), BARD1 E386X (0.035), KMT2A K1029Rfs*65 (0.034), ARID1A S2262X (0.033), FGF1 P42L (0.029)*	
#31	iCCA	64	f	absent	MSI-	FGFR2 C383R (0.856)	FGFR2 C382R (0.289), *PALB2 K268N (0.071)*	
#32	iCCA	68	f	absent	MSI-	FGFR2-SLMAP (F17;S3) (373)	FGFR2-SLMAP (2211), **BAP1 R57Pfs*14 (0.605), TSC1 L250X (0.519), MLH1 K732E (0.518), TSC2 K347R (0.501),** MLLT3 S190_T191insSSSS (0.370)**,** EP300 N452S (0.224), *NOTCH3 P1521del (0.069),* MDM4 amplification (fold change 2.263)	

#33	iCCA	67	f	absent	MSI-	IDH1 R132L (0.422)	**APC S2258A (0.431),** PIK3CD F698Cfs*2 (0.379), IDH1 R132L (0.310), CCNE1 amplification (fold change 2.446)	
#34	iCCA	62	m	absent	MSI-	KRAS Q61H (0.570)	**CCND3 R167W (0.463)**, AR Q80_E81insQQ (0.383), TP53 K101X (0.348), RAF1 L613V (0.301), *PIK3CA R87T (0.079)* , *ROS1 C89W (0.65), FOXL2 A14G (0.052),* MYC amplification (fold change 2.128), FGFR1 amplification (fold change 2.046), FGF19 amplification (fold change 2.042)	KRAS Q61H was not identified by targeted DNA sequencing because of low coverage
#35	iCCA	27	f	absent	MSI-	BRAF V600E (0.182)	**ERBB4 R983I (0.743)**, BRAF V600E (0.257), CARD11 K911Q (0.244), *NPM1 D180del (0.058)*	
#36	iCCA	55	f	absent	MSI-	IDH1 R132C (0.059)	**ROS1 T2039M (0.487)**, ARID1A Y1324X (0.102), *FBXW7 splice site c.585-2->T (0.051), IDH1 R132C (0.044)*	
#37	iCCA	63	f	absent	MSI-	IDH1 R132C (0.223)	NBN I171V (0.329), BAP1 L112Qfs*13 (0.191), IDH1 R132C (0.168)	
#38	iCCA	60	f	absent	MSI-	IDH1 R132C (0.659)	**RPS6KB1 G504R (0.591), APC E1317Q (0.572), CREBBP V2048M (0.492), CHEK2 S5L (0.479),** PTCH1 V1034M (0.410), ALK V1039M (0.362), IDH1 R132C (0.297), KRAS amplification (fold change 3.286)	
#39	iCCA	55	f	nd	MSI-	IDH R132C (0.214)	**MRE11 Q509Pfs*5 (0.668)**, IDH1 R132C (0.115)	
#40	iCCA	55	m	absent	MSI-	WT	**BRCA2 R2336H (0,444; ClinVar: Pathogenic)**, ARID1A Y1052C (0.310), *BRCA1 P773Lfs*17 (0.09),* MDM2 amplification (fold change 6.584), KRAS amplification (fold change 4.367)	

#41	iCCA	69	m	absent	MSI-	FGFR2-BICC1 (F17;B3) (6404)	FGFR2-BICC1 (8869), **MLLT3-CNTLN (22 reads),** BAP1 E20del (0.167)	
#42	iCCA	62	m	absent	MSI-	WT	**KDR E749K (0.475), BRCA2 A1170S (0.463), FGFR3 E368K (0.414),** *GNAQ Y101X (0.092)*	The variant of unknown significance in FGFR3 gene could be missed by targeted RNA sequencing because of low FGFR3 expression
#43	iCCA	34	m	absent	MSI-	PIK3CA I1062F (0.338)	ARID1A I908N (0.366), TP53 V272E (0.339), PIK3CA I1062F (0.276), MAP2K1 Q56P (0.1), MYC amplification (fold change 4.037)	
#44	iCCA	68	f	absent	MSI-	FGFR2-AHCYL1 (F17;A2) (24)	FGFR2-AHCYL1 (59)	
#45	iCCA	68	m	absent	MSI-	WT	**BAP1 Y646X (0.764), ARID1A K1094Sfs*67 (0.677), MAP2K1 Y130C (0.527), CDK12 L666I (0.451), TSC2 K347R (0.433)**, KIT R946X (0.389), *APC E2184K (0.057)*	
#46	iCCA	51	f	absent	nd	FGFR2-VCL (F17;V2) (2489)	FGFR2-VCL (6905), MDM2 amplification (fold change 4.469)	
#47	iCCA	33	f	absent	MSI-	FGFR2-BICC1 (F17;B3) (3804) + I548V (0.522)	FGFR2-BICC1 (10670) + I547V (0.230), BAP1 splice site c.122+1G>C (0.269)	

[1]The number in brackets shows the count of supporting reads for translocations and the fraction of an alternate allele for other mutations; the results of DNA sequencing with mutant allele fraction more than 0.4 (possible germline variants) are shown in **bold**, while low-prevalent (subclonal) variants are shown in *italic* ; the discrepant cases are marked by red font.
